# Single-Molecule Localization Microscopy Reconstruction Using Noise2Noise For Super-Resolution Imaging of Actin Filaments

**DOI:** 10.1109/ISBI45749.2020.9098713

**Published:** 2020-05-22

**Authors:** Joël Lefebvre, Avelino Javer, Mariia Dmitrieva, Jens Rittscher, Bohdan Lewków, Edward Allgeyer, George Sirinakis, Daniel St. Johnston

**Affiliations:** Big Data Institute, Institute of Biomedical Engineering, University of Oxford, UK; Big Data Institute, Institute of Biomedical Engineering, University of Oxford, UK; Big Data Institute, Institute of Biomedical Engineering, University of Oxford, UK; Big Data Institute, Institute of Biomedical Engineering, University of Oxford, UK; St Johnston Lab, Wellcome Trust / CRUK Gurdon Institute, University of Cambridge, UK; St Johnston Lab, Wellcome Trust / CRUK Gurdon Institute, University of Cambridge, UK; St Johnston Lab, Wellcome Trust / CRUK Gurdon Institute, University of Cambridge, UK; St Johnston Lab, Wellcome Trust / CRUK Gurdon Institute, University of Cambridge, UK

**Keywords:** Single-Molecule Localization Microscopy, Image Reconstruction, Self-supervision, Actin

## Abstract

Single-molecule localization microscopy (SMLM) is a super-resolution imaging technique developed to image structures smaller than the diffraction limit. This modality results in sparse and non-uniform sets of localized blinks that need to be reconstructed to obtain a super-resolution representation of a tissue. In this paper, we explore the use of the Noise2Noise (N2N) paradigm to reconstruct the SMLM images. Noise2Noise is an image denoising technique where a neural network is trained with only pairs of noisy realizations of the data instead of using pairs of noisy/clean images, as performed with Noise2Clean (N2C). Here we have adapted Noise2Noise to the 2D SMLM reconstruction problem, exploring different pair creation strategies (fixed and dynamic). The approach was applied to synthetic data and to real 2D SMLM data of actin filaments. This revealed that N2N can achieve reconstruction performances close to the Noise2Clean training strategy, without having access to the super-resolution images. This could open the way to further improvement in SMLM acquisition speed and reconstruction performance.

## 1 Introduction

SMLM is a super-resolution imaging technique developed to visualize structures smaller than the diffraction limit. Many SMLM modalities exist[1], including peptide-PAINT. This approach uses short-peptide strands functionalized with fluorescent dyes that selectively bind to biological structures of interest. The linked peptide then emits short bursts of photons that appear as isolated blinks on the recording camera. The sub-pixel positions of the blinks can be extracted using a variety of fitting methods[2]. Blinking positions are accumulated until enough events are collected to visualize the tissue structure. PAINT-based imaging relies on sparse blinking density to achieve precise localization.

A recent technique, ANNA-PALM [3], has tackled the SMLM reconstruction problem where a clean image needs to be obtained from a non-uniform and sparse set of localized blinks. It uses an artificial neural network to reconstruct super-resolution representations of microtubules, nuclear pores, and mitochondria using two orders of magnitude less data than the conventional methods. The authors used a convolutional neural network (CNN) that was trained in a fully supervised setting (Noise2Clean, N2C). The training task was to predict the super-resolution reconstruction image from a small fraction of the localization data. Although the authors of this paper have shown that ANNA-PALM provides significant improvements in acquisition speed and reconstruction quality, the acquisition of high-density data is not always possible. An alternative to the N2C approach is Noise2Noise (N2N) [4]. In this configuration, a denoising network is trained by only using noisy image pairs of the same underlying signal. The authors have shown that given enough iterations, networks trained with N2N will learn to restore the clean images. Their method achieved close to state-of-the-art denoising performance without ever using clean images that are often hard or impossible to obtain.

In this paper, we explore the use of the Noise2Noise paradigm to tackle the SMLM reconstruction problem in a 2D setting using only sparse localization data. We have implemented the N2N method using a CNN inspired by U-Net [5], an encoder-decoder network with skip connections. We have investigated two approaches to generate pairs of sparse images: fixed and dynamic, where the fixed pairs are generated prior to training, and the dynamic pairs are generated on-the-fly during training. The technique was tested using both synthetic data and 2D SMLM data of actin filaments. Our experiments indicate that the N2N approach achieves results close to those obtained with the N2C training strategy, while it doesn’t require the super-resolution images to train the reconstruction model.

## 2 Methodology

### 2.1 SMLM Reconstruction

The reconstruction model is based on Noise2Noise [4]. Briefly, lets consider pairs of sparse images (*x*_*i*,1_, *x*_*i*,2_) obtained from two random subsets *A*_*i*,*k*_ selected from the set *B*_*i*_ of all localized blink positions for a given SMLM experiment. These subsets are mutually exclusive, such that *A*_*i*,1_ ∩ *A*_*i*,2_ ≡ ∅, and *A*_*i*,1_ ∪ *A*_*i*,2_ ∈ *B*_*i*_. Then the N2N learning task consists in minimizing the following equation:

(1) $$\underset{\theta }{\text{argmin}}\underset{i}{\sum \;}L\left(f_{\theta }\left(x_{i,1}\right),x_{i,2}\right),$$

where *f*_*θ*_ is the reconstruction model with parameters *θ*, and *L* is a loss function. The original N2N paper has shown that given enough data and for unclipped Gaussian noise, the minimization converges to the unobserved clean target *y*_*i*_. This training approach was also shown experimentally to give good results even for other noise distributions, such as Poisson noise, multiplicative Bernoulli noise and others. For the SMLM problem, the reconstruction uncertainty comes from blink localization errors, from non-uniform sampling, and from data sparsity.

Here, we used a U-Net inspired architecture as the reconstruction model [5]. In this network, each convolution is followed by a leaky ReLU with a negative slope of 0.1, and without any batch normalization. All the convolutions use a 3x3 kernel except for the initial block where a 7x7 kernel is used. The downstream encoder consists of four blocks each with two convolutions with 48 filters followed by a max pooling. The upstream decoder consists of four blocks each with a 2x upsampling by nearest neighbours and the corresponding downstream block concatenation followed by two convolutions with 48 filters. Finally, the output is reduced to one channel using a convolution with linear activation. We used the smooth L1 loss (Huber loss) as the minimization criterion, wherein a L2 or L1 loss is used based on the element-wise error magnitude. This loss was chosen because it is less sensitive to outliers, while retaining the L2 criterion properties for small magnitude element-wise errors. To train the network, we used the Adam optimizer with learning rate = 1e-4 and weight decay = 0.0. The batch size was 128, and the training patch size was 64x64 pixels. The training was performed until a total of 1M samples were presented to the network, using epoch size of 100k samples.

### 2.2 Synthetic Data Generation

**Fig. 1:**
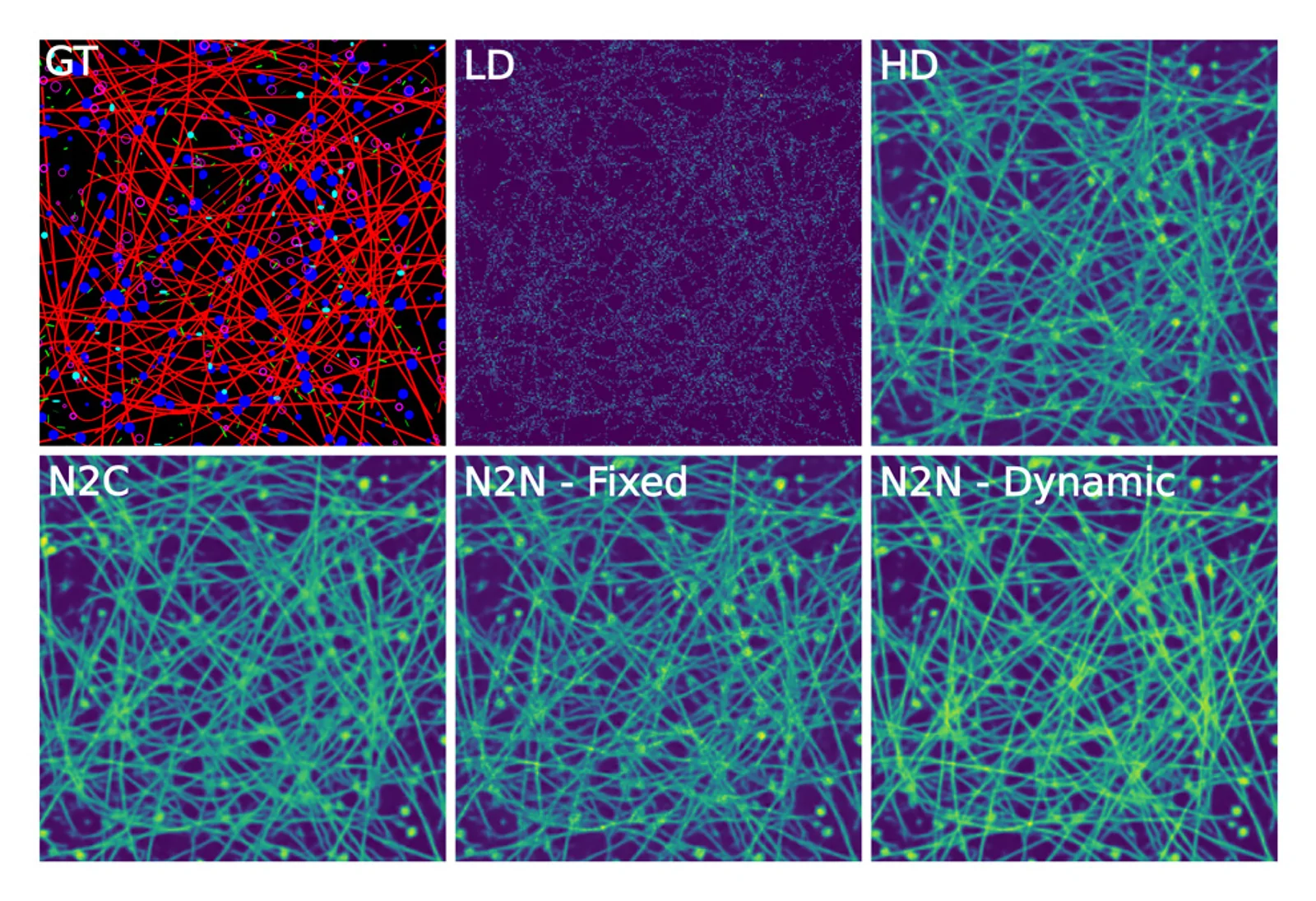
Synthetic data reconstruction with various training strategies. GT: ground truth with color-coded geometrical primitives, LD: Low density, HD: High density, N2C: Noise2Clean, N2N - Fixed: N2N with fixed image pairs, N2N - Dynamic: N2N with dynamic image pairs.

To develop and validate the N2N-based reconstruction method, we created a simple 2D SMLM simulator akin to one used for the 3D SMLM localization challenge [2]. The simulator is organized in three steps. First, a set of geometrical primitives (B-splines, lines, circles, ellipses and rings) are generated by randomly selecting their parameters (e.g. position, size, thickness, etc.). Then, an image of the underlying ground truth structure is reconstructed from the primitives given a field of view size and a simulation resolution (*r* = 2 *nm*). The third step is blink position generation. This is done by randomly selecting emitter positions within the ground truth image, and by modifying the blink position by a random Gaussian noise representing the localization error. Figure 1 represents a synthetic 2D SMLM image generated with our 2D simulator.

### 2.3 Super-resolution Acquisitions of Actin

We have also applied the N2N-based reconstruction to real 2D SMLM data. In this study we used fluorescently labelled LifeAct (Cy3b-MGVADLIKKFESISKEE-acid) as a peptide-PAINT probe for visualization of the f-actin structures in Drosophila follicular epithelium. LifeAct is a short 17-amino acid peptide that transiently binds f-actin structures with a high specificity [6]. In order to visualize the actin cytoskeleton, we fixed and permeabilized Drosophila ovaries (W^1118^ flies) and mounted them on microscope slides with the labelling solution. The flies were fattened two days before dissection and kept at 25°C. Dissection of the ovaries was performed directly into 38°C warm Fixation Buffer (4% formaldehyde, 2% Tween 20 in Hypotonic Buffer). Samples were fixed for 20 min at room temperature with rotation. Later, samples were washed and permeabilized 3x 10min with 0.2% PBST. The ovaries were dissected on the slide, muscle sheet was separated from egg chambers and an excess of water was removed using pipette and tissue. Approximately 40*µl* of Labelling Solution (5-10nM LifeAct in PBS with 1% Catalase and 0.25% Glucose Oxidase) was added on top of egg chambers. Microscope slides were sealed with spacers in between using two-compound silicone glue and kept in the dark for 5-10 min until the glue solidified.

All actin super-resolution imaging was performed on a custom built confocal slit scanning microscope centered around an Olympus IX-83 microscope base. In brief, 546 nm CW laser light was coupled into a single mode optical fiber. Light emitted from the fiber was collimated and directed through a cylindrical lens subsequently focusing the light into a line on a galvo scanning mirror conjugated to the back pupil plane of an objective lens (Olympus, 100X UPlanSApo, 1.35NA, silicon immersion). At the sample, the line of focused excitation laser light was diffraction limited in one dimension and 20 *µm* in length in the orthogonal direction. The galvo mirror scanned the excitation line across the sample at a rate corresponding to the camera frame rate. Fluorescence emission was collected by the same objective lens and imaged onto an sCMOS camera with a 256 x 256 image format and an effective pixel size of 98 nm. The employed field of view was 20 x 20 *µm*. As the excitation line was scanned across the sample, the sCMOS camera (Hamamatsu, Orca Flash 4.0 V2) was operated in so-called light-sheet mode to create an effective electronic confocal slit. Thus, out of focus background was reduced. Camera frames were acquired and subsequently analyzed with previously published software tools to localize the blinks positions to be used for the N2N reconstruction [7].

### 2.4 Training Approaches

We experimented with various training approaches to investigate their effect on the reconstruction performance. We have tested 3 data pairs creation methods: (1) low and high density image pairs (N2C), (2) fixed low density image pairs (*n2n-fixed*), and (3) dynamic low density image pairs (*n2n-dynamic*). The difference between the fixed and dynamic pairs is that the blinks positions are either used to create pairs of images before training, or used to create new pairs of images during training at each iteration. For *n2n-dynamic*, at each iteration the blink positions were augmented using random crops, rotations, and vertical and horizontal flips on the blink localization space. The resulting transformed coordinates were then randomly sampled to only keep 95% of the data. The image pairs were generated by randomly splitting the data in two subsets and assigning each blink to its closest neighbour in a 10 nm resolution grid. The reconstruction performance was evaluated with the structural similarity (SSIM) and the peak signal-to-noise ratio (PSNR) between the reconstructed image and its high-density counterpart. The clean images were synthesized with the simulator by setting blink density to a much higher value (2*M* blinks/ *µm*^2^) compared to the low density regime (10*k* blinks/ *µm*^2^). Also, to enable the comparison between fixed and dynamic image pairs, the blink positions that were generated by the simulator for *n2n-fixed* were joined into a single point-cloud that was used by *n2n-dynamic* during training.

## 3 Results and Discussion

### 3.1 Evaluation on synthetic data

**Fig. 2:**
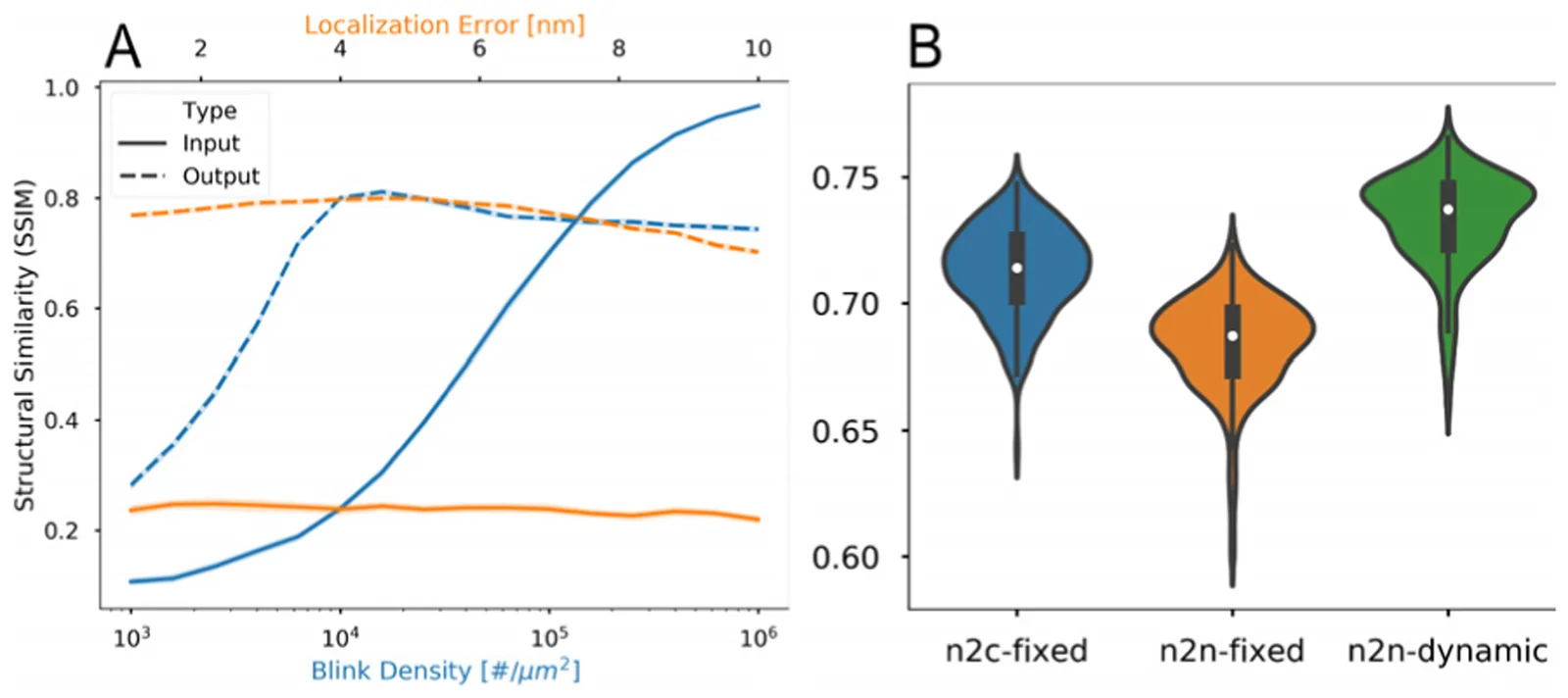
Reconstruction performances with the synthetic data. (A) Changes in SSIM with different localization errors *σ* and blink densities *d* for a model trained with *σ* = 5 *nm* and *d* = 10*k* blinks/ *µm*^2^, (B) Effect of the training strategy on the reconstruction performance (SSIM).

The first reconstruction experiments were performed with the 2D SMLM simulator directly. The simulator was integrated into the training loop to generate new pairs of synthetic images on-the-fly for every learning iteration. This effectively replicates the behaviour of *n2n-dynamic* with a large dataset. We trained the reconstruction network by setting the localization error to 5 nm and the blink density to 10*k* blinks/ *µm*^2^. We then tested the reconstruction performance with the same simulator, but by generating new images with varying blink densities and localization errors (Fig 2A). This analysis shows that the *n2n-dynamic* approach is able to reconstruct good estimates of the high density image with less data. Both SSIM and PSNR increase as the input image density increases, until it reaches the same blink density as the one used for training. The reconstruction quality then stabilizes (SSIM at 0.8 and PSNR between 17 and 18) for larger blink densities^1^. The reconstructed images are only surpassed by the input data when it reaches blink densities above 100k/FOV. This is the level above which both the SSIM and PSNR of the input images are higher than the reconstructed images from these inputs when compared to the target. This indicates that for the synthetic data, the N2N paradigm can achieve similar performances with one order of magnitude less data (10k vs. 100k detections per FOVs to predict the high density image). For the localization error, the SSIM metric is maximum when reconstructing images generated with the same localization error as the ones used during training, although this effect is smaller than blink density effect on reconstruction performance. This indicates that the reconstruction method and the blink localization algorithm have orthogonal effects on the super-resolution reconstruction quality. Future developments to further improve SMLM image reconstruction should thus consider both the localization algorithms and the reconstruction methods in an integrated framework.

The second experiment with the synthetic data was performed to evaluate the effect of the training approach (N2C vs. N2N) and the effect of the data pairs type (fixed vs. dynamic) on the reconstruction performance. This experiment was performed with a dataset of 64 synthetic images of size 256x256px generated with our custom simulator. All other model and training parameters were kept the same between experimental units. To evaluate the effect of selection bias with this data, a 4-fold cross-validation with random selection was performed for each training configuration. The training and testing losses were evaluated after the optimization. This revealed low variance between the CV folds for both losses (between 0.8% and 2.5%), indicating a low selection bias for the synthetic dataset. The reconstruction was then evaluated on a separate set of validation images (N=64) and is reported in Fig2B. This revealed that *n2n-fixed* leads to lower SSIM values than the N2C approach, but *n2n-dynamic* leads to an increased SSIM compared to the supervised method. To explain this effect, we hypothesize that the dynamic pair generation acts as an additional regularization that creates smoother reconstruction compared to directly learning a mapping from sparse to dense images. For this experiment, the training approaches did not result in statistically different PSNR values (tested with 2-sample Student’s T-test with *α* = 0.005).

### 3.2 Evaluation with 2D actin data

**Fig. 3:**
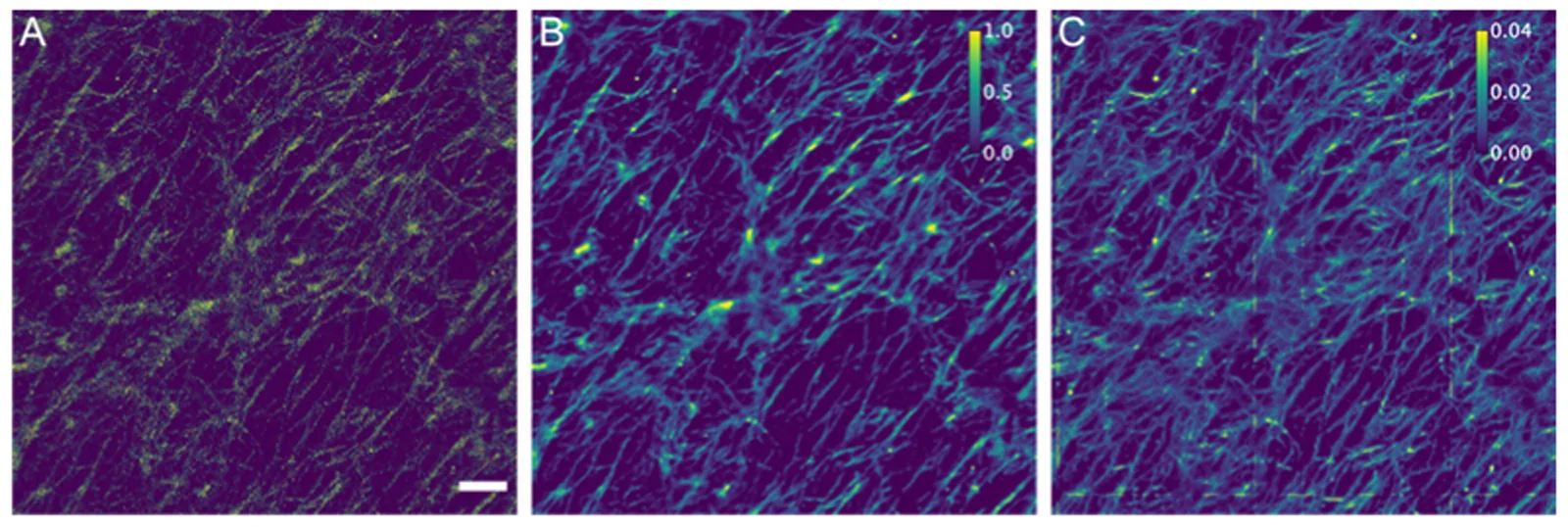
Example of actin image reconstruction: sparse input (A), the average reconstruction (B) and the average absolute deviation from the mean reconstruction (C). Note that the intensity calibration bars are different between B and C, and that the scale bar is of size 1 *µm*.

Lastly, our reconstruction method using N2N with dynamic pairs generation was tested with a real actin dataset to show that this technique can be used in an experimental setting. The dataset consisted in the localized blinks positions for 10 peptide-PAINT acquisitions of fixed actin filaments in developing Drosophila eggs, as summarized in the section 2.3. The average blink density for this dataset was (1.9 ± 0.35) × 10^3^ blinks/ *µm*^2^, and the reconstruction resolution was set to 10 nm. We trained the U-Net using 10-fold cross-validation with random subsets. We then evaluated the reconstruction performance on an additional validation image that was not used during training (Fig3). As the blink density was 5x smaller for the actin dataset compared to the synthetic data, this resulted in loss curves that were significantly noisier. Despite this, the deviations of each image from the average reconstruction was on average less than 10% of the average pixel intensity, and this deviation affected mostly the image texture and not the underlying reconstructed actin filaments structure. Thus, using self-ensembling during inference and reducing texture variability with additional regularization or using different reconstruction model could be beneficial in future improvements of this method.

## Conclusion

We have investigated the use of the N2N paradigm to reconstruct super-resolution images from 2D SMLM datasets. Our experiments showed that N2N can achieve similar or better reconstruction performances than the N2C supervised method. This is a promising preliminary result, and thus N2N for SMLM merits further investigations. Some important topics that could be addressed in future works are: exploring the generalizability and transferability of the method, evaluating the influence of the CNN model architecture on the reconstruction, and extending the N2N to 3D data.

## Footnotes

1 Only SSIM is illustrated in the figure, as PSNR exhibited similar trends
